# Calcitonin substitution in calcitonin deficiency reduces particle-induced osteolysis

**DOI:** 10.1186/1471-2474-12-186

**Published:** 2011-08-15

**Authors:** Max D Kauther, Hagen S Bachmann, Laura Neuerburg, Martina Broecker-Preuss, Gero Hilken, Florian Grabellus, Gabriele Koehler, Marius von Knoch, Christian Wedemeyer

**Affiliations:** 1 Department of Trauma Surgery, University Duisburg-Essen, Hufelandstr. 55, 45147 Essen, Germany; 2 Department of Orthopaedics, University Duisburg-Essen, Hufelandstr. 55, 45147 Essen, Germany; 3 Institute of Pharmacogenetics, University Duisburg-Essen, Hufelandstr. 55, 45147 Essen, Germany; 4 Department of Endocrinology, Division of Clinical Chemistry and Laboratory Medicine, University Duisburg-Essen, Hufelandstr. 55, 45122 Essen, Germany; 5 Central Animal Laboratory, University of Duisburg-Essen, Hufelandstr. 55, 45122 Essen, Germany; 6 Institute of Pathology and Neuropathology, University of Duisburg-Essen, Hufelandstraße 55, 45122 Essen, Germany; 7 Institute of Pathology, University of Münster, Domagkstraße 5, 48149 Münster, Germany; 8 Department of Orthopaedic Surgery, Klinikum Bremerhaven, Postbrookstr. 103, 27574 Bremerhaven, Germany

## Abstract

**Background:**

Periprosthetic osteolysis is a major cause of aseptic loosening in joint arthroplasty. This study investigates the impact of CT (calcitonin) deficiency and CT substitution under in-vivo circumstances on particle-induced osteolysis in *Calca *-/- mice.

**Methods:**

We used the murine calvarial osteolysis model based on ultra-high molecular weight polyethylene (UHMWPE) particles in 10 C57BL/6J wild-type (WT) mice and twenty *Calca *-/- mice. The mice were divided into six groups: WT without UHMWPE particles (Group 1), WT with UHMWPE particles (Group 2), *Calca *-/- mice without UHMWPE particles (Group 3), *Calca *-/- mice with UHMWPE particles (Group 4), *Calca *-/- mice without UHMWPE particles and calcitonin substitution (Group 5), and *Calca *-/- mice with UHMWPE particle implantation and calcitonin substitution (Group 6). Analytes were extracted from serum and urine. Bone resorption was measured by bone histomorphometry. The number of osteoclasts was determined by counting the tartrate-resistant acid phosphatase (TRACP) + cells.

**Results:**

Bone resorption was significantly increased in *Calca *-/- mice compared with their corresponding WT. The eroded surface in *Calca *-/- mice with particle implantation was reduced by 20.6% after CT substitution. Osteoclast numbers were significantly increased in *Calca *-/- mice after particle implantation. Serum OPG (osteoprotegerin) increased significantly after CT substitution.

**Conclusions:**

As anticipated, *Calca *-/- mice show extensive osteolysis compared with wild-type mice, and CT substitution reduces particle-induced osteolysis.

## Background

Periprosthetic osteolysis is known to be a major cause of aseptic loosening in joint arthroplasty [1]. This process is mainly mediated by wear particles that stimulate an inflammatory response in the surrounding tissue, leading to implant loosening. As a result, challenging revision surgery becomes necessary [2]. The neurotransmitter calcitonin gene-related peptide (CGRP) has been found in the synovial fluid and neocapsules of patients with loosened implants, and it appears to play an important role in neuro-osteogenic interactions [3,4]. Wedemeyer et al. recently evaluated a genetically modified mouse strain with alpha-CGRP-gene deficiency in order to gain a better understanding of the alpha-CGRP-mediated effect in particle-induced osteolysis. They observed a decrease in ultra-high molecular weight polyethylene (UHMWPE) particle-induced osteolysis in alpha-CGRP deficient mice compared to their corresponding wild-type mice (WT), which led them to conclude that the fine tuning of osteoclasts mediating resorption in alpha-CGRP-null mice may be deregulated [5]. Alpha-CGRP is expressed in the cells of the central and peripheral nervous system and is mostly known for its regulatory effect on the vascular system [6,7] and its inhibition of the differentiation and recruitment of osteoclast precursors. However, the expression of the CGRP-receptor on primary osteoclasts has a modulatory function on osteoclasts by a direct mechanism [8,9]. Alpha-CGRP is generated from the *Calca *gene that further codes for calcitonin (CT) by cell-specific alternative splicing [10,11]. Since the discovery of CT in the 1960s, it has been known to have an inhibitory effect on osteoclast activity [11]. More recently, Gooi et al. described a negative influence of CT on bone formation [12]. Calcitonin is mainly produced in the thyroidal C cells and is a single-chain peptide of 32 amino acids in length with a molecular mass of 3,418 daltons and a half-life in serum of approximately ten minutes [13]. Substitution of calcitonin leads to mild hypocalcaemia and hyperphosphataemia through an inhibitory effect on osteoclast activity [11,14]. Salmon CT is preferred as a therapeutic agent against Paget's disease and osteoporosis because of its ability to slow down bone resorption by inhibiting osteoclast activity, even though calcitonin is not widely used to treat osteoporosis [15-17]. Both CT and alpha-CGRP inhibit bone resorption [18,19]. In this manuscript we are following up our previous study on alpha-CGRP deficiency in which we analyzed the influence of CT on particle-induced osteolysis [5]. Hoff et al. stated that "the absence of the *CT/CGRP *gene is associated with increased bone formation not only in the basal state, but also in a condition characterized by increased osteoclastic resorption" [20]. They described an unexpected phenotype of high bone mass in *Calca *deficiency that was contrary to the expected effects of both alpha-CGRP and CT [20]. The increase in bone formation accompanied by an increase in bone resorption raises the question of the consequences on UHMWPE particles. We hypothesized that Calcitonin substitution might inhibit particle-induced osteolysis and that *Calca *deficient mice might develop a greater extent of osteolysis compared to the WT. Our recent study revealed an unexpected decrease in particle-induced osteolysis in alpha-CGRP deficient animals, therefore we were especially interested in the *Calca *deficient mice. This study investigates the impact of CT deficiency and CT substitution on particle-induced osteolysis.

## Methods

### Animals

The animal experiment was approved by the institutional animal care and oversight committee. We used a calvarial model of UHMWPE particle-induced osteolysis in ten twelve-week old male C57BL/6J mice obtained from Jackson Laboratories (Bar Harbor, Main, USA) and twenty male *Calca *deficient mice of the same age with a background of C57BL/6. The mice were kept in a climate-controlled room (22°C; 45-54% relative humidity) with a 12-hour light/12-hour dark cycle. Food and water were given *ad libitum*. The animals were divided into six groups of five mice. C57BL/6J mice, wild-type mice (WT), were treated in Groups 1 and 2. *Calca *deficient mice were used in Groups 3, 4, 5, and 6. Groups 1, 3, and 5 underwent sham surgery only. Groups 2, 4, and 6 received UHMWPE particles. Groups 5 and 6 received additional, daily s.c. injections of 2 μg human-CT (Sigma Aldrich, T3535, Saint Louis, Missouri, USA) in the dorsal neck, dissolved in 250 μL 0.9% saline, for the full duration of the experiment (14 days) as described by Kapurniotu et al. [21]. Table 1 shows the six different groups.

**Table 1:** Study design of the 6 different groups

	WT	Calca -/-	Calca -/- + CT
Sham	Group 1	Group 3	Group 5

UHMWPE	Group 2	Group 4	Group 6

Each group consisted of 5 mice.

### Particles

The Clariant Company (Gersthofen, Germany) supplied the commercially pure UHMWPE particles (Ceridust VP 3610). More than 35% of the particles were smaller than 1 μm, with a mean particle size (given as equivalent circle diameter) of 1.75 ± 1.43 μm (range 0.05-11.6) [22]. For decontamination of endotoxins, the particles were washed twice in 70% ethanol at room temperature for 24 hours. The particles were then washed in phosphate-buffered saline and dried in a desiccator. The particles were stored in endotoxin free, sterile tubes. Testing for endotoxins was performed using a quantitative Limulus Amebocyte Lysate (LAL) Assay (Charles River, Margate, U.K.) whereby the detection level of < 0.25 EU/ml was set as negative.

### Surgical Procedure

The mice were anaesthetized by intraperitoneal injection with 80 mg/kg ketamine (CEVA, Sante animale Ketaminhydrochlorid, Düsseldorf, Germany) and 10 mg/kg xylazine (CEVA). A 10 mm incision was made over the calvarian sagittal midline suture. A 1.0 × 1.0 cm area of periosteum was exposed and left intact. In the sham controls (Groups 1, 3, and 5) the incision was closed without any further intervention. Groups 2, 4, and 6 received 30 μl of dried polyethylene particles (2 × 10^8 ^particles per 1000 μl; the dosage was established through previous experiments [5,23]) which were distributed over the periosteum using a sterile sharp surgical spoon. The incision was sutured. Fourteen days after operation the animals were sacrificed in a CO_2 _chamber, as our previous research had shown that particle-induced osteolysis can be detected from this time point [5,23].

### Serum and Urine Analyses

Preoperatively, blood samples of 250 μl were obtained by orbita puncture and urine was collected in sterile tubes. Further blood samples were taken by means of cardiac puncture before sacrifice. The serum samples were centrifuged at 3000 × g for ten minutes, aliquoted and frozen at -70°C. Total calcium, alkaline phosphatase and inorganic phosphorus in the serum were measured using a RX Monza analyzer (Randox, Antrim, UK) with suitable reagents and controls (Randox, Antrim, UK). Creatinine, total calcium and inorganic phosphorus in the urine samples were measured within two hours after collection using an ADVIA 2400 instrument (Siemens Medical Solutions, Fernwald, Germany) and suitable reagents and controls (Siemens Medical Solutions, Fernwald, Germany). Serum osteoprotegerin (OPG) and TNF-related activin-induced cytokine (TRANCE, RANKL) were determined by Immunoassays (Quantikine assays, R&D Systems, Minneapolis, MN, USA). Mouse osteocalcin (OCN) was determined using an IRMA kit purchased from Immutopics (San Clemente, CA, USA). Deoxypyridinoline (DPD) crosslinks in urine were measured using the DPD EIA kit (Quidel, San Diego, CA, USA). DPD values were normalized to the urinary creatinine concentration and expressed as nmol DPD/mmol creatinine. All assays were performed according to the manufacturer's instructions.

### Bone Histomorphometry

The calvaria were removed as an elliptical plate of bone defined by the foramen magnum, auditory canals, and orbits [24]. Hair and brain tissue were eliminated. The calvaria were decalcified and cut into four pieces vertical to the midline suture, oriented on edge, and embedded in paraffin blocks. The embedded tissues were cut into 4- μm sections in the coronal plane using a microtome. Specimens were photographed digitally with a standard high-quality light microscope at a magnification of x10 and x20 with the midline suture in the middle of the field. Histomorphometric analysis was performed using image analysis software (UTHSCA Image Tool, IT version 3.0; University of Texas, San Antonio, TX). To determine bone thickness, specimens were divided into four steps, each of 0.5 mm width, to the left and four steps, again each of 0.5 mm width, to the right side of the midline suture. The mean bone thickness for each group was calculated successively from the means of these nine regional bone thickness measurements. The area of soft tissue including any bone resorption pits in the midline suture was traced in hematoxylin and eosin-stained sections to determine the bone resorption area in the midline suture [24,25]. The non-osseous tissue area adjacent to and in continuity with the midline suture was encircled independently by two of the authors. Within this field, the number of osteoclasts per bone perimeter was determined. Osteoclasts were identified as large multinucleated TRACP (tartrate-resistant acid phosphatase) + cells located on the bone perimeter. The values of each available section were averaged per animal and the assessed values for bone resorption area and the number of osteoclasts were averaged again for each group.

### Statistical Analysis

Data are reported as mean ± SD. The results were analyzed by two-way analysis of variance (ANOVA) to show an influence of particles (main effect), group (main effect) or the combination of particles and group (interaction effect). As we observed evidence for the interaction of almost all outcome measures, we performed two-sided Student's t-test for independent samples to analyze the difference of the means between pairs of groups (Groups 1-6 as shown in Table 1). Student's t-Test was also used to determine differences of the preoperative biochemical analysis between WT and *Calca *deficient mice. We applied a significance level α of 5% (two-sided).

## Results

### Serum and Urine Analyses

**Figure 1:**
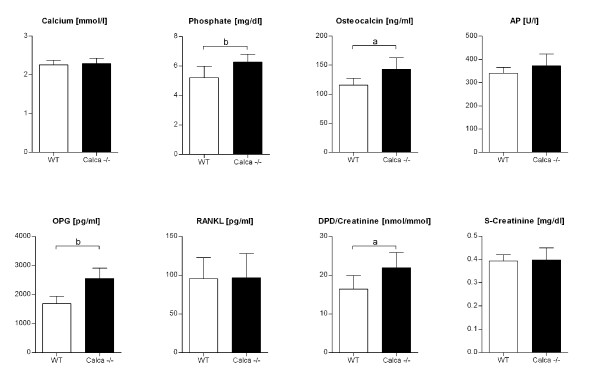
**Preoperative serum and urine levels of WT and *Calca *-/- mice**. Differences were found in OCN, DPD/creatinine, OPG, and phosphate. The data are expressed as mean ± SD. (a: p < 0.01, b: p < 0.001)

Biochemical analysis revealed differences in the phenotype of C57BL/6J (n = 10) and *Calca *knockout mice (n = 20). Preoperatively, significantly higher levels of OCN (p < 0.01), DPD/creatinine (p < 0.01), OPG (p = 0.0000002), and phosphate (p < 0.001) were found in the *Calca *-/- mice compared to the wild-type (Figure 1). A postoperative decrease in OCN and an increase in calcium, phosphate, and DPD/creatinine were found in all groups compared with the preoperative levels. DPD/creatinine levels revealed significant differences in osteolysis between Groups 1 and 2 (p < 0.05), though there were no significant differences between Groups 3 and 4. Group 5 showed significantly higher (p < 0.05) DPD/creatinine levels compared to the particle-treated Group 6. Analysis of the postoperative serum parameters demonstrated significant inter-group differences, but the change in the serum and urine parameters could not be correlated with the change in BV/TV or the eroded surface. Remarkably, postoperative OPG was not significantly different between Groups 1 and 2 (1631 ± 190 pg/ml vs. 1654 ± 199 pg/ml) nor between Groups 3 and 4 (2336 ± 290 pg/ml vs. 2432 ± 476 pg/ml), but a significant difference was detected for Group 5 vs. Group 6 (2342 ± 237 pg/ml vs. 3298 ± 699 pg/ml; p < 0.05).

### Bone Histomorphometry

**Figure 2:**
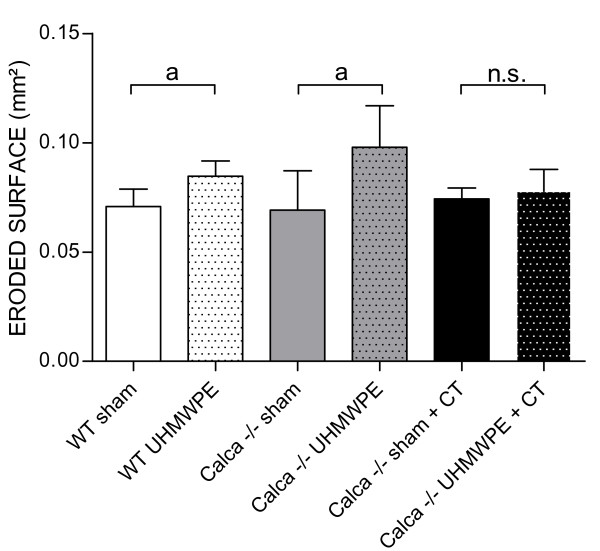
**Eroded bone surface surrounding the midline suture determined by histomorphometry 14 days postoperatively**. ANOVA testing indicated a significant influence of particles (p = 0.001) and the combination of particles and group (p < 0.05). The data are expressed as mean ± SD (a: p < 0.05, b: p < 0.01).

**Figure 3:**
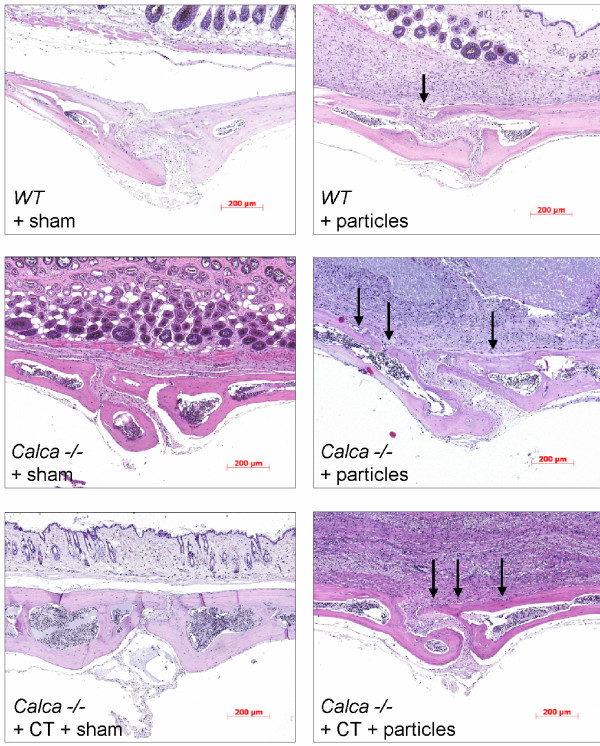
**Histomorphometrical comparison of particle-induced osteolysis in WT mice and *Calca -/- *mice 14 days after surgery**. Representative hematoxylin and eosin-stained sections are shown. The midline suture is in the middle of the section. The arrows mark osteolytic defects that are mostly found centrally in the area of the midline suture.

ANOVA testing of the eroded surface in continuity to the midline suture showed a significant influence of particles (p = 0.001) and of the combination of particles and group (p < 0.05 for the interaction term; Figure 2; Figure 3). The eroded surface in Group 1 was found to be 0.0708 ± 0.008 mm^2 ^(range 0.0605 - 0.0794 mm^2^) compared to 0.0847 ± 0.007 mm^2 ^(range 0.0797 - 0.0969 mm^2^) in Group 2 (p < 0.05). Bone resorption in Group 3 was 0.0692 ± 0.018 mm^2 ^(range 0.0339 - 0.0860 mm^2^) compared to 0.0980 ± 0.011 mm^2 ^(range 0.0791 - 0.1279 mm^2^) in Group 4 (p < 0.05). Bone resorption in Group 5 (0.0743 ± 0.005 mm^2^; range 0.0684 - 0.0828 mm^2^) did not show significant differences compared to Group 6 (0.0778 ± 0.010 mm^2^; range 0.0641 - 0.0876 mm^2^). The eroded surface was significantly reduced by 20.6% after CT substitution of *Calca *deficient mice with particle implantation (Group 4 vs. Group 6) (p < 0.05).

**Figure 4:**
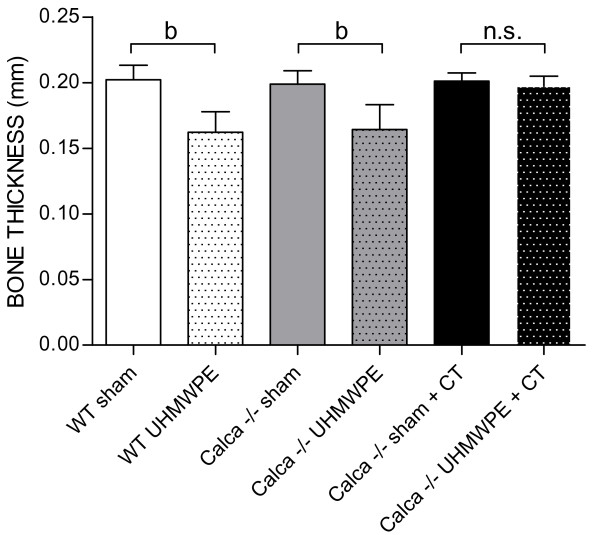
**Average bone thickness determined by histomorphometry 14 days postoperatively**. ANOVA testing indicated a significant influence of group (p < 0.05) and the combination of particles and group (p < 0.001). The data are expressed as mean ± SD (a: p < 0.05, b: p < 0.01).

ANOVA testing of bone thickness indicated a significant influence of group (p < 0.05) and the combination of particles and group (p < 0.001; Figure 4). Further testing showed significant differences between Group 1 and Group 2 (202 μm ± 11 μm vs. 162 μm ± 15 μm; p < 0.05) and significant differences between Group 3 and Group 4 (198 μm ± 10 μm vs. 164 μm ± 19 μm; p < 0.01). Particles in the calcitonin substituted Groups 5 and 6 did not lead to a significant change in bone thickness (201 μm ± 6 μm vs. 197 μm ± 8 μm; (p = 0.39)).

### Osteoclasts per Bone Perimeter

**Figure 5:**
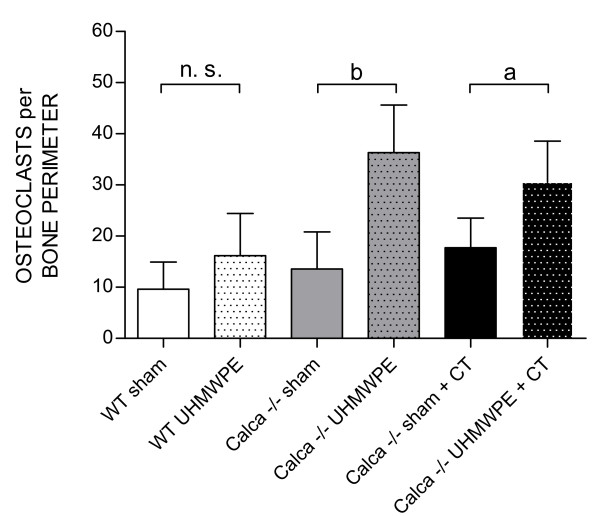
**Average number of osteoclasts per bone perimeter within the different subgroups at 14 days postoperatively**. ANOVA testing indicated a significant effect of group (p < 0.01) and particles (p < 0.001). For each group, the data are expressed as mean ± SD (a: p < 0.05, b: p < 0.01).

**Figure 6:**
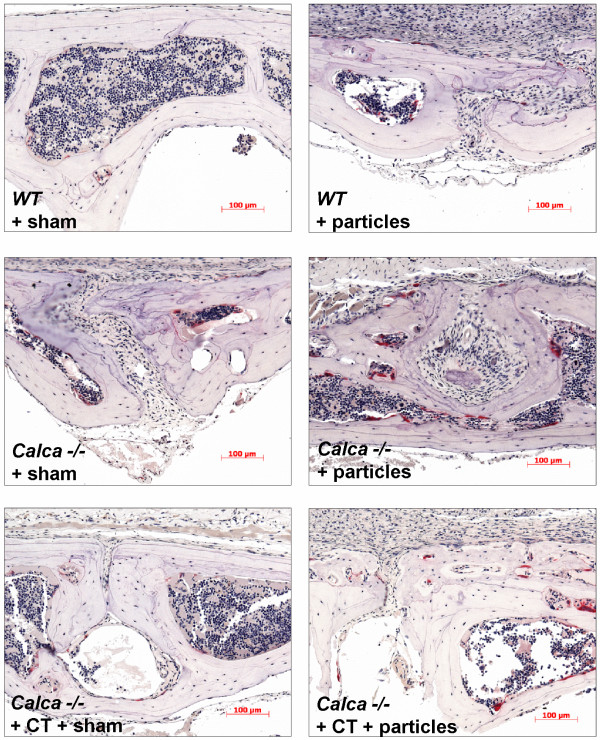
**Histomorphometrical comparison of osteoclasts in WT mice and *Calca *-/- mice 14 days after surgery**. TRACP immunohistochemistry shows purple stained TRACP + positive osteoclasts.

ANOVA testing of the number of osteoclasts indicated a significant effect of group (p < 0.01) and particles (p < 0.001; Figure 5; Figure 6) whereas the combination of particles and group yielded a p = 0.067 which, though not significant, prompted us to perform t-test comparisons of the groups as before. The number of osteoclasts per bone perimeter in Group 1 was found to be 9.65 ± 5.26 (range 0 - 22) compared to 16.17 ± 8.26 (range 6 - 43) in Group 2 (p = 0.17). The number of osteoclasts in Group 3 was 13.58 ± 7.23 (range 6 - 35) compared to 36.35 ± 9.29 (range 7 - 53) in Group 4 (p < 0.01), and in Group 5 it was 17.72 ± 5.78 (range 7 - 35) compared to 35.55 ± 8.04 (range 7 - 43) in Group 6 (p < 0.05).

## Discussion

Aseptic loosening is caused by wear particles which stimulate an inflammatory response mediated by macrophages and giant cells. Furthermore, polyethylene particles can induce a catabolic phenotype of osteoblasts which also contribute to implant loosening [26]. Since the histological studies on loose hip prostheses by Ahmed et al. [4] and Saxler et al. [27] there has been much discussion about a possible influence of the nervous system on particle-induced osteolysis. These authors found sensory nerve fibers in the surrounding granulomatous tissue which led them to hypothesize that there may be a link between the nervous system and particle-induced osteolysis. This thesis was further supported by the discovery of elevated CGRP and Substance P in synovial fluid from patients with loose implants and by our previous studies on alpha-CGRP and Substance P knockout mice showing a significant influence on particle-induced osteolysis [3,5,23]. Both alpha-CGRP and calcitonin are generated by alternative splicing from the *Calca *gene, therefore we were interested in the outstanding phenotype of *Calca *deficient mice with an increased bone formation rate (BFR) in contrast to the decreased BFR in alpha-CGRP knockout mice [20,28]. Hoff et al. stated that the observed increase in bone mass in the KO mice results from an increase in bone formation rather than a decrease in osteoclast resorption at the age of 1 and 3 months [20]. Huebner et al. found a significant increase in bone volume and an up to four-fold elevation of osteoclast numbers in *Calca *^-/- ^mice at the age of 12 months compared to wild-type or alpha-CGRP^-/- ^mice [28]. As CT has been used to treat metabolic bone disease with decreased osteoclastic function, it is of great interest to gain a better understanding of the effects of CT and its interaction with alpha-CGRP in particle-induced osteolysis under in-vivo circumstances.

The laboratory results of 20 *Calca *-/- mice revealed a high bone turnover similar to that of the *Calca *-/- phenotype previously described by Huebner et al. [28,29]. As far as we are aware, this is the largest description of clinical serum chemistry involving *Calca *-/- mice at the age of three months. DPD, a marker for bone resorption, OCN, a marker for osteoblast activity, and OPG, an osteoprotective transmitter were significantly elevated compared to the WT background of C57BL/6J. These results correspond to those of Hoff et al. who found significantly elevated DPD levels at the age of one month, while no significant differences could be detected at three months. This may have been due to a high standard deviation in the five animals they analyzed [20]. Unfortunately, the extent of postoperative osteolysis did not correlate with any of the analyzed serum parameters. We recently described DPD as a possible marker to detect particle-induced osteolysis [30], yet despite this finding, it could not be correlated with the extent of osteolysis in *Calca *-/- mice in this study, although the largest extent of osteolysis was found in these groups. Further biochemical markers should be analyzed for detection of particle-induced osteolysis. In the present study *Calca *-/- animals showed significantly pronounced osteolysis after UHMWPE particle implantation in comparison with their corresponding WT, a finding which was confirmed by histomorphometry. Taking the results of our previous study on alpha-CGRP deficient mice into consideration, the bone resorption mediated by particle-induced osteolysis in mice with *Calca *deficiency was significantly greater in extent than the osteolysis seen in alpha-CGRP knockout mice and their corresponding WT [5]. As such a large extent of osteolysis was not seen in the particle-treated *Calca *-/- group (Group 6) that received daily substitution of CT we assume that artificial CT substitution might be able to compensate the CT knockout and lead to a reduced osteolytic reaction. It is possible that CT may have an even more potent effect on particle-induced osteolysis than alpha-CGRP, a theory that has also been reported by other authors [31]. Thus, the *Calca *-/- mice that received additional CT substitution during the experimental setup showed a phenotype similar to the phenotype seen in alpha-CGRP deficient animals [5]. Neither in the *alpha-CGRP *deficient mice, nor in the *Calca *-/- mice with additional CT substitution was the effect of particle-induced osteolysis significant. Thus, the osteo-anabolic effect of artificial CT substitution could be comparable to the intranasal substitution of CT to prevent osteoporosis [17]. We believe that our findings underline the theory of an inhibitory effect of CT on bone resorption [18]. The differences in resorption seen in histomorphometry could be explained by a deregulation of osteoblasts and osteoclasts or their interaction with each other. CT is known to inhibit osteoclast differentiation and bone resorption under in-vitro and in-vivo circumstances [32]. The direct influence of CT on osteoclasts is mediated by the CT receptor which is localized on the surface of osteoclasts [33]. Thus, CT binds to the CT-receptor leading to reduced production of proteolytic enzymes and a decrease in osteoclast formation on the osseous surface. It was therefore not surprising that the *Calca *-/- animals that received UHMWPE particles showed the greatest accumulation of osteoclasts associated with the greatest extent of osteolysis. The *Calca *-/- animals treated with UHMWPE particles and artificial CT substitution also showed significantly more osteoclasts than their corresponding control group. However, the effect appeared to be less pronounced. The detected DPD serum levels were significantly higher in *Calca *-/- animals than in C57BL/6J mice, which could be an expression of higher bone resorption. These changes might contribute to a combined activity of osteoblasts and osteoclasts, since OPG as an osteoprotective transmitter was also found to be significantly increased in *Calca *-/- mice compared to WT mice.

Despite the known influence of CT on osteoclasts, there is only little evidence to support the theory that there is a CT receptor on the surface of osteoblasts [34,35]. Hoff et al. discussed unknown pathways of CT which could also induce an indirect activation of osteoblasts [20]. Gooi et al. recently described the CT-receptor on osteocytes with a negative regulation on bone formation [12]. Lymphocytes that express the CT receptor might also be able to stimulate bone remodeling by means of the transcription factors Cbfal and OPG [36]. We were able to confirm the possibility of this pathway, especially for OPG, by serum analysis in the present study. OPG serum levels were significantly elevated in the *Calca *-/- animals and artificial CT substitution leads to an additional significant elevation of OPG serum levels in particle- and CT-treated *Calca *-/- mice postoperatively. We analyzed the OCN levels further as an indicator of osteoblast activity. The significantly elevated OCN levels assessed preoperatively indicate an increased osteoblast activity in *Calca *-/- animals. However, CT substitution did not cause an increase in OCN serum levels. Moreover, the postoperative OCN levels were significantly reduced in all *Calca *-/- mice by between 15 to 18% compared to the preoperative serum levels. Another experimental setup to further analyze the observed effects of CT in the absence of alpha-CGRP could be an investigation on genetically modified animals with a CT receptor (CTR) knockout. There have already been investigations using a CTR haploinsufficient animal model with a CTR expression decreased by 50%, as homoinsufficiency was lethal. The study by Dacquin et al. observed increased bone formation with no consequences for bone resorption [37]. Another CTR knockout was introduced by Davey et al. using the Cre-*lox*P system, in which the CTR is globally deleted by > 94% but < 100% [38]. The authors reported that these CTR knockouts displayed normal serum ultrafiltrable calcium levels and a mild increase in bone formation in males, showing that CTR plays a modest physiological role in the regulation of bone and calcium homeostasis in the basal state in mice.

The limitations of our study should be taken into account. The model employed in our investigation uses a flat bone that is formed by membranous rather than endochondral ossification. The comparison of the WT animals and *Calca *deficient mice must be treated with caution because we used animals from different animal facilities with possible mutations of their common C57BL/6 background. The daily injection of calcitonin in Groups 5 and 6 might have had an influence on local tissue reaction or systemic inflammation. As we did not employ a control group the reaction off a vehicle or placebo is unknown. Nevertheless, calcitonin led to decreased osteolysis, whereas a local or systemic inflammation due to injections would possibly have lead to increased osteolysis. It is known that endotoxins lead to encreased osteolysis in the calvarial model [39]. Furthermore, particle-induced osteolysis must be regarded as a multifactor process. *Calca *deficiency might influence other aspects of bone metabolism.

Although it can be assumed that there are interactions mediated by neurotransmitters in particle-induced osteolysis in KO animals, the impact of the genetic background in humans remains unclear as aseptic loosening occurs at a different time in life and after a different postoperative period in each individual patient. Wedemeyer et al. therefore recently analyzed single nucleotide polymorphisms of the human *Calca *gene, but were not able to detect a significant correlation with aseptic loosening in their preliminary study [40].

## Conclusions

The unexpected finding of increased osteolysis in the *Calca -/- *mice and the expected reduction in osteolysis in *Calca *-/- mice due to CT substitution suggest that CT has an osteoprotective impact on particle-induced osteolysis. Calcitonin appears to have a stronger effect on osteoclasts than on osteoblasts in particle-induced osteolysis in Calca -/- mice. These findings might be helpful for the prevention of aseptic loosening and could offer further treatment options.

## List of Abbreviations

BV: bone volume; CGRP: calcitonin gene-related peptide; CT: calcitonin; DPD: deoxypyridinoline; LAL: Limulus Amebocyte Lysate; μ-CT: micro-computed tomography; OCN: osteocalcin; OPG: osteoprotegerin; ROI: Region of Interest; TRACP: tartrate-resistant acid phosphatase; TRANCE: TNF-related activin-induced cytokine; UHMWPE: ultra-high molecular weight polyethylene; WT: wild-type; CTR: calcitonin receptor

## Competing interests

The authors declare that they have no competing interests.

## Authors' contributions

MDK, CW and MvK designed and coordinated the study, carried out the animal experiments, and drafted the manuscript. HSB participated in the design of the study and performed the statistical analysis. LN and MBP analyzed the serum and urine samples and drafted the manuscript. GH coordinated and carried out the animal experiments. FG and GK carried out the histological preparations and stainings. All authors read and approved the final manuscript.

## Pre-publication history

The pre-publication history for this paper can be accessed here:

http://www.biomedcentral.com/1471-2474/12/186/prepub
